# What Factors Contribute to Postabortion Contraceptive Uptake By Young Women? A Program Evaluation in 10 Countries in Asia and sub-Saharan Africa

**DOI:** 10.9745/GHSP-D-17-00085

**Published:** 2017-12-28

**Authors:** Janie Benson, Kathryn Andersen, Joan Healy, Dalia Brahmi

**Affiliations:** a Ipas, Chapel Hill, NC, USA.

## Abstract

**Background:**

Unintended pregnancy disproportionately affects young women and adolescents in developing countries. The abortion care setting offers a unique opportunity for adolescents and young women to access a full range of contraceptive services. This evaluation assesses the factors that influence contraceptive uptake among adolescents and young women seeking abortion care in health facilities.

**Methods:**

Following provider training, we analyzed client log book data from 921,918 abortion care cases in 4,881 health facilities in 10 countries from July 2011 through June 2015. Log book data included client characteristics such as age, pregnancy gestation, type of service provided, and contraceptive method provision. Health facility characteristics were obtained through administration of a site baseline form prior to initiation of programmatic support by Ipas, an international NGO. Programmatic support included integration of postabortion contraceptive services with abortion care, improvements in commodities logistics, health worker training, upgraded recordkeeping, and post-training follow-up with providers and sites to solve problems and improve performance. We analyzed abortion cases by 3 age categories, ≤19 years, 20–24 years, and ≥25 years, and conducted unadjusted and adjusted analyses for the primary outcomes of interest: receipt of a contraceptive method at the time of care; type of contraceptive method selected; and the client, clinical care, and facility characteristics associated with contraceptive uptake.

**Results:**

Overall, 77% of women left the facility with a contraceptive method. The majority (84%) of contraceptive acceptors selected a short-acting method, especially oral contraceptives. In the adjusted model, women ≤19 were less likely to choose a method than women 25 years or older (odds ratio [OR], 0.87; 95% confidence interval [CI], 0.79 to 0.96). Adolescents and young women were also significantly less likely to choose a long-acting, reversible contraceptive than those ages 25 or older (≤19 years: OR, 0.59; 95% CI, 0.52 to 0.67; 20–24 years: OR, 0.68; 95% CI, 0.63 to 0.73). Women treated by an Ipas-trained provider were significantly more likely to select postabortion contraception than women treated by non-Ipas-trained providers (OR, 1.37; 95% CI, 1.20 to 1.57).

**Conclusions:**

Programmatic support to health systems, including provider training in contraceptive counseling and provision, was associated with women's higher acceptance of postabortion contraception. However, gaps remained for young women, especially adolescents, who were significantly less likely than older women to accept postabortion contraception. Health systems and facilities should pay increased attention to meeting the contraceptive needs of young women and adolescents.

## Abstract (precis)

Across the 10 countries, 77% of 921,918 women left with a contraceptive method after receiving abortion care. While contraceptive uptake was high among all age groups, adolescents ages 15–19 were less likely to choose a method than women 25 years or older.

## INTRODUCTION

About one-half of pregnancies to adolescents 15–19 years of age are unintended and about one-half of these end in abortion.^1^ Adolescents and young women seeking abortion are more likely to obtain care at later gestations in their pregnancies, use less-skilled practitioners, and experience delays in care when complications occur.^1^^–^^3^

Unsafe abortion disproportionately affects adolescents and young women in developing countries. Between 2010 and 2014, almost 25.1 million unsafe abortions occurred globally each year, most in developing countries.^4^ Adolescents aged 15–19 and young women 20–24 comprised an estimated 41% of unsafe abortions globally in 2008, and in Africa about one-half of unsafe abortions were among these age groups.^5^

Unsafe abortion disproportionately affects adolescents and young women in developing countries.

Underlying these findings are low and/or inconsistent use of contraception among adolescents and young women leading to unintended pregnancies. An estimated 38 million adolescents 15–19 years of age in developing countries are married or unmarried and sexually active and do not wish to have a child within the next 2 years, yet only 40% are using a modern method of contraception.^1^ The most commonly used method is male condoms, comprising 38% of methods used by adolescent acceptors; condoms require partner collaboration and suffer from higher failure rates than other modern methods. An analysis of contraceptive use and discontinuation in more than 40 countries found that failure rates were about 25% higher within the first year of method use among 15–19-year-old adolescents compared with women 20 or older.^6^ Use of modern contraception among married women 20–24 years of age in least-developed countries is 29%.^7^

Abortion care services include provision of induced abortion as well as treatment of incomplete abortion resulting from complications of unsafe abortion or spontaneous abortion; the latter is referred to as postabortion care (PAC). Abortion care services also include postabortion contraceptive counseling and method provision. The terms “induced abortion” and “PAC treatment” are used in this article if a particular setting or finding refers to one or the other type of service. The abortion care setting offers a unique opportunity for adolescents and women to access contraceptive information and methods, if they wish to use one. They are present in a health facility and may not return for subsequent care. Ovulation returns quickly after an abortion and contraception can enable women to avert an unintended pregnancy.^8^^,^^9^ For women who wish to become pregnant again following a spontaneous abortion, current evidence recommends 6-month spacing of the subsequent pregnancy for optimal outcomes.^10^

The abortion care setting offers a unique opportunity for adolescents and women to access contraceptive information and methods, if they wish to use one.

Multiple studies have demonstrated the effectiveness of interventions to provide contraceptive counseling and methods to abortion clients prior to discharge from health facilities.^11^^–^^14^ Most have demonstrated high uptake of methods at the time of services, although fewer studies have assessed contraceptive continuation and longer-term reproductive outcomes. Assessments of postabortion contraceptive interventions to reach young women and adolescents obtaining abortion care have also reported the effectiveness of contraceptive care offered at the time of the abortion.^15^^–^^17^

Only a few studies have assessed the client, clinical care, and institutional factors that affect women's acceptance of postabortion contraception.^13^^,^^18^^,^^19^ The analysis described in this article examines the factors that are associated with postabortion contraceptive uptake among young women and adolescents so that health systems can use the findings to strengthen services for this population. While some countries have limitations on contraceptive provision to young women and/or unmarried women, many are increasing women's awareness of contraceptive options, strengthening providers' skills, and expanding access to long-acting, reversible contraceptives (LARCs) as part of commitments made at the 2012 London Summit on Family Planning.^20^

This article examines the factors associated with postabortion contraceptive uptake among young women and adolescents.

We report results of programmatic support to health facilities in 10 countries that aimed to improve provision of contraception to women seeking induced abortion or PAC treatment. Support was provided by Ipas, an international NGO, and its network partner, the Ipas Development Foundation, a local NGO in India, in collaboration with ministries of health and other local entities. The countries in our analysis were Ghana, Nigeria, Sierra Leone, Uganda, and Zambia in sub-Saharan Africa, and Bangladesh, India, Myanmar, Nepal, and Pakistan in Asia.

Objectives of this article are to: (1) describe an intervention aimed at improving postabortion contraceptive counseling and method provision; (2) assess the factors that influence contraceptive uptake among clients at the time of abortion care, by age; and (3) recommend steps to improve the quality of postabortion contraceptive services in health facilities for young women and adolescents.

## PROGRAM DESCRIPTION

The type of abortion care provided in facilities varied by the legal, policy, and cultural contexts of the 10 countries. In Nepal, for example, most clients sought an induced abortion, while in Pakistan, Sierra Leone, and Uganda, most women sought PAC treatment of incomplete abortion. In other countries, such as Bangladesh and India, both induced abortion and PAC cases were included in the analysis. Postabortion contraceptive counseling and methods were incorporated into both types of abortion care.

### Postabortion Contraceptive Support

Postabortion contraceptive support to health systems and facilities fell into 9 domains:

1. Development of technical standards, guidelines, and protocols for provision of care to all women, regardless of age, marital status, or other barriers, based on World Health Organization (WHO) guidance
2. Physical upgrades in facilities to facilitate contraceptive availability at the time of abortion care
3. Improvements in logistics processes for commodities
4. Training-of-trainers and in-service training of physicians, midwives, clinical officers, and other cadres in clinical care, including contraceptive counseling and provision
5. Values-clarification training for service providers and staff to reduce stigma toward women and adolescents who need reproductive health services
6. Provision of contraceptive counseling and methods at the same location and time as abortion care
7. Separate, specialized training of providers in counseling and provision of available LARC methods in selected countries, including on medical eligibility, method side effects, counseling approaches at the time of abortion care and afterward, and method insertion
8. Improvements in abortion case recordkeeping and regular use of data to monitor service availability and quality
9. Routine follow-up of facilities and trained providers to identify and resolve barriers to care.

In most countries, providers received periodic in-person visits and phone check-ins from Ipas clinical mentors, program staff, and/or health systems supervisors following training, along with a review of their individual summary monitoring data, which underscored contraception as a routine component of comprehensive care.

Clinical training courses for providers usually lasted 5 days, but in some countries were held for 10–12 days. The courses were led by clinical master trainers who had previously completed training-of-trainers courses in abortion care. Training participants included obstetrics and gynecology specialists, general physicians, midwives, clinical officers, nurses, and other cadres, depending on the country setting. Follow-up contacts with trained providers usually occurred within 3–4 weeks post-training, either by phone or in-person at their facility. During the check-ins, the mentor/program support specialist assessed the newly trained provider, identified and resolved clinical or programmatic concerns, reviewed log book records (if in-person), and agreed to a follow-up contact time. Subsequent support visits from the program support team member also included review of individualized performance data with the provider. Frequently addressed issues included stock-outs of commodities, need for additional clinical practice, reinforcement of infection prevention practices, insufficient support of upgraded care by facility management, and low caseloads. Examples of strategies to resolve these problems included making contacts with supply chain officials at the facility or higher level; discussions with department heads, facility administrators, and district supervisors on the importance of the service; use of clinical practice job aids; and expanded outreach to inform the community about the service.

In-person support to trained providers occurred at their health facility, as noted, but often included interactions and refresher sessions with providers who did not participate in the training but were part of the larger team of caregivers. Technical assistance to and monitoring of health facilities was also carried out, recognizing that providers require a supportive environment in which to practice.

While programmatic support in all countries encompassed core training curricula, standard log book formats, and monitoring tools, each program was designed and implemented to reflect local service delivery contexts, including compliance with Ministry of Health standards and guidelines. Women selected from the abortion methods available in the facility and for which they were medically eligible. Contraceptive commodities included only those methods currently approved and available in each country's health system. The timing of contraceptive counseling, prior to or after the abortion procedure, also varied by the client's clinical condition, type of contraceptive methods available in the setting, and other factors. Ipas does not recommend the inclusion of clients' marital status in abortion care log books since this information can be used to deny women care. However, the Ministries of Health of some countries required this information to be recorded. Client exit interviews were conducted annually in selected facilities in many of the countries to gather women's perspectives on the quality of information that providers shared during their visit, provision of women's preferred contraceptive methods, and source of referral to the facility. Despite these programmatic differences between countries and changes over time, implementation of the core Ipas training curriculum for development of providers' clinical and counseling skills was relatively standardized but updated as new, published evidence became available.^21^ WHO recommendations and new findings from scientific literature, including provision of hormonal methods on the first day of a medical abortion, were incorporated into training although their integration into country-level standards and guidelines varied.^21^^,^^22^ More details on Ipas programmatic support have been published elsewhere.^18^

### Support for Reaching Young Women and Adolescents With Postabortion Contraception

Programmatic support for reaching young women and adolescents was carried out in all countries but varied by the specific context. Program components included assessments of existing youth-focused services in health facilities to assist in program planning, upgrades to sites to improve client privacy and confidentiality, provider training on clinical and counseling issues relevant to young clients, sensitization of facility managers and health officials to the needs of young women, and an array of community outreach activities for young women and adolescents. Counseling training focused on a woman-centered approach to identify and address clients' individual needs and preferences in preventing pregnancy and providing voluntary, informed consent. Trainers in India, Nepal, Nigeria, and Zambia also received in-depth skills training on rights-based approaches to contraception. Some programs began as pilot projects in a few health facilities and were subsequently expanded in several countries to broader health systems efforts. Ipas resources such as a training toolkit for abortion care for young women helped guide the design and implementation of programmatic support.^23^

## METHODS

### Data Collection and Processing

Data were collected from abortion care log books in public- and private-sector facilities in the 10 countries. Intervention sites were selected by Ipas and collaborating partners, based on geographic priorities for coverage, Ministry of Health strategies, weak reproductive health indicators, potential for integration of a new/upgraded service, and other criteria. Individual case information on abortion care clients was recorded by providers over a 4-year period from July 2011 through June 2015, and collected by Ipas during monitoring visits to the sites; frequency of the visits varied by country but usually occurred monthly, quarterly, or every 6 months. Collection of monitoring data began after provider training due to the poor quality of log books prior to start-up of the intervention. Client names, their record numbers, or other identifying data were not collected.

Abortion care log books were part of the routine health recordkeeping system and included client characteristics such as age, pregnancy gestation (in weeks), and type of service (induced abortion, PAC, or other); the treatment regimen such as the method used for the abortion (manual or electric vacuum aspiration [MVA/EVA], medical abortion, or other), pain management, and contraceptive method provided at the time of care, by method type; adverse events; and provider signature or identification. While Ipas recommends that providers indicate women's desire for contraception, in addition to whether they received a method at the time of care, most health systems did not include this information in log books. Many abortion care providers had participated in an Ipas-supported in-service clinical training and were assigned a unique provider ID. During training, providers were instructed on how to complete the log book and asked to add their signature to cases they treated. Log book cases with a signed entry were assumed to have been attended by a provider who had participated in an Ipas-supported training. Information about clients treated in the facility by providers not trained by Ipas were also included in the dataset. Information about facility type, level, and other characteristics was obtained on a site baseline form completed by Ipas before initiation of programmatic support.

All log book and site data were entered into Ipas's programmatic monitoring database and cleaned and analyzed using Stata version 11.0. The protocol was approved by the Allendale Institutional Review Board (US).

### Data Analysis

We analyzed abortion cases by 3 age categories: ≤19 years, 20–24 years, and ≥25 years. Primary outcomes of interest included: receipt of a contraceptive method at the time of care; type of contraceptive method selected; and the client, clinical care, and facility characteristics associated with contraceptive uptake within and across each age category.

Rates of missing data on age were low, 2% overall; these cases were removed from the dataset. We treated data conservatively, classifying those cases without contraceptive information in the log books as not having received a method. The abortion care delivery setting for each client age category was described by region, health sector (public or private facility), level of the facility (primary, secondary, or tertiary), and whether the health care worker who offered the abortion participated in an Ipas-supported training. Additional client data included gestational age, type of abortion (induced abortion or PAC treatment), and the abortion method used.

We conducted unadjusted and adjusted analyses for the outcomes of interest. For each age group, we describe overall contraceptive uptake; type of contraceptive method such as condoms, oral contraceptives, or intrauterine devices (IUDs); and method category (short-acting or LARC). Short-acting methods consisted of condoms, oral contraceptives, and injectables. LARC methods were implants and IUDs. The percentage of clients receiving a sterilization procedure was low across all age groups (7%) and not included in our analysis. Percentages are reported for non-missing data.

Bivariate analysis of overall contraceptive uptake and by method category was carried out with facility and clinical characteristics. Chi-square tests were applied to determine statistical significance. Adjusted analysis was conducted using logistic regression, controlling for facility clustering and country-level variations. We conducted tests of collinearity and model fit to select the most parsimonious model. Significance levels were set at 0.05.

## RESULTS

### Background Characteristics

Findings are based on 921,918 abortion cases treated in 4,881 health facilities: India (3,178 health facilities); Nigeria (464); Nepal (383); Ghana (278); Pakistan (200); Bangladesh (175); Zambia (104); Uganda (48); Myanmar (37); and Sierra Leone (14). A large majority of cases, 67%, occurred in sites in Asia (Table 1). Clients receiving abortion care in India (41%), Nigeria (18%), and Bangladesh (17%) made up more than three-quarters of all cases. In total, 7% of clients were 19 years old or younger and 31% were 20–24 years old.

**TABLE 1.** Background Characteristics of Abortion Clients Treated in Health Facilities in 10 Countries, by Client Age, 2011–2015

	Age Group (in years)			Total (N=921,918) No. (%)
	≤19 (n=68,265) No. (%)	20–24 (n=282,695) No. (%)	≥25 (n=570,958) No. (%)	
Region				
Africa	43,741 (64)	86,356 (31)	170,101 (30)	300,198 (33)
Asia	24,524 (36)	196,339 (69)	400,857 (70)	621,720 (67)
Country				
India	12,351 (18)	140,765 (50)	227,526 (40)	380,642 (41)
Nigeria	18,639 (27)	45,592 (16)	94,631 (17)	158,862 (18)
Bangladesh	7,959 (12)	35,162 (12)	112,872 (20)	155,993 (17)
Ghana	13,534 (20)	21,990 (8)	42,639 (7)	78,163 (8)
Zambia	10,221 (15)	17,301 (6)	30,981 (5)	58,503 (6)
Nepal	3,352 (5)	14,144 (5)	36,914 (6)	54,410 (6)
Pakistan	697 (1)	5,437 (2)	20,234 (4)	26,368 (3)
Myanmar	165 (<1)	831 (<1)	3,311 (1)	4,307 (<1)
Uganda	844 (1)	1,006 (<1)	1,359 (<1)	3,209 (<1)
Sierra Leone	503 (1)	467 (<1)	491 (<1)	1,461 (<1)
Facility sector^a^				
Private	18,062 (26)	72,249 (26)	142,644 (25)	232,955 (25)
Public	50,203 (74)	210,446 (74)	428,314 (75)	688,963 (75)
Facility level^b^				
Primary	30,014 (44)	135,047 (48)	295,074 (52)	460,135 (50)
Secondary	30,239 (44)	115,472 (41)	212,371 (37)	358,082 (39)
Tertiary	8,012 (12)	32,176 (11)	63,513 (11)	103,701 (11)
Ipas-trained provider				
Yes	43,373 (64)	165,235 (58)	342,928 (60)	551,536 (60)
No	24,892 (36)	117,460 (42)	228,030 (40)	370,382 (40)
Gestational age, weeks^c^				
≤12 weeks	59,940 (91)	257,377 (95)	525,011 (95)	842,328 (95)
≥13 weeks	5,657 (9)	12,927 (5)	25,087 (5)	43,671 (5)
Missing	2,668	12,391	20,860	35,919
Type of abortion^c^				
Induced abortion	35,712 (55)	155,123 (59)	327,872 (60)	518,707 (60)
Postabortion care	29,374 (45)	107,331 (41)	215,776 (40)	352,481 (40)
Missing	3,179	20,241	27,310	50,730
Abortion method^c^				
MVA/EVA	48,400 (71)	202,512 (73)	413,550 (75)	664,462 (74)
Medical abortion	16,241 (24)	58,114 (21)	113,812 (21)	188,167 (21)
D&C/D&E/Other	3,086 (5)	15,488 (6)	24,530 (4)	43,104 (5)
Missing	538	6,581	19,066	26,185

Abbreviations: D&C, dilation and curettage; D&E, dilation and evacuation; EVA, electric vacuum aspiration; MVA, manual vacuum aspiration.

a NGO-sponsored facilities were recategorized as public or private based on country context.

b Approximately 6% of cases in India and Uganda were served in facilities that were categorized as “other”; these were recategorized as “primary.”

c Missing data were not included in the calculation of percentages.

The women served in Africa were younger than those in Asia. Almost two-thirds (64%) of women 19 years and under were from Africa while 36% were from Asia. Most (95%) women sought care during the first trimester of pregnancy, but 9% of clients age 19 or younger obtained care at 13 weeks of pregnancy or later compared with 5% each of women ages 20–24 and those ages 25 and older. In addition, a higher percentage of adolescents ages 15–19 received PAC treatment (45%) than women ages 25 or older (40%). Service delivery characteristics varied little by age. Most (89%) clients were seen in primary- or secondary-level public facilities, and Ipas-trained providers attended 60% of the clients. Almost three-quarters of cases were performed with vacuum aspiration (MVA or EVA).

### Postabortion Contraceptive Uptake

Overall contraceptive uptake was high for all age categories in the unadjusted analysis, with 77% of women leaving the health facility with a contraceptive method. Highest uptake was among women ≥25 years of age (78%), and lowest among women under age 20 (71%) (Table 2).

**TABLE 2.** Contraceptive Uptake at the Time of Abortion Care Among Clients in 10 Countries, by Client Age, 2011–2015

	Age Group (in years)			Total (N=921,918) No. (%)
	≤19 (n=68,265) No. (%)	20–24 (n=282,695) No. (%)	≥25 (n=570,958) No. (%)	
Any uptake of postabortion contraception				
Yes	48,725 (71)	215,964 (76)	444,503 (78)	709,192 (77)
No	13,948 (20)	39,631 (14)	77,857 (14)	131,436 (14)
Missing	5,592 (8)	27,100 (10)	48,598 (9)	81,290 (9)
Method category				
Short-acting	42,104 (86)	186,685 (86)	366,709 (82)	595,498 (84)
Long-acting reversible	6,621 (14)	29,279 (14)	77,794 (18)	113,694 (16)
Method type				
Condoms	13,142 (27)	60,007 (28)	92,072 (21)	165,221 (23)
Oral contraceptive pills	16,900 (35)	95,821 (44)	194,932 (44)	307,653 (43)
Injectables	12,062 (25)	30,857 (14)	79,705 (18)	122,624 (17)
Implants	4,471 (9)	7,669 (4)	20,296 (5)	32,436 (5)
Intrauterine device	2,150 (4)	21,610 (10)	57,498 (13)	81,258 (11)

77% of women left the health facility with a contraceptive method.

Of those clients who chose contraception, 84% received a short-acting method. This did not vary markedly across age groups: a slightly higher percentage of women 25 and older (18%) selected a LARC method compared with those under 25 (14%) (Table 2). The specific types of methods chosen followed similar trends across the age groups: oral contraceptive pills were the most commonly selected method for each age group, followed by condoms and injectables. The least commonly chosen method was the IUD for women under 20 and implants for women in the other age groups. Of note, a lower percentage of women under 20 chose pills than women 20 or older (35% vs. 44%, respectively), and a higher percentage of women under 20 chose injectables than women 20 or older (25% vs. 14%–18%, respectively) (Table 2).

Oral contraceptive pills were the most commonly selected method for each age group, followed by condoms and injectables.

#### Factors Associated With Postabortion Contraceptive Uptake

Factors positively associated with postabortion contraceptive uptake among women of all ages included obtaining services in a private facility, receiving care from a provider trained as part of an Ipas-supported intervention, being ages 25 years or older, first-trimester gestational age, seeking an induced abortion (compared with receiving PAC services), and choosing a medical abortion. In the adjusted analysis, facility sector was not significantly associated with postabortion contraceptive acceptance, and women under age 20 were less likely to choose a contraceptive method than women 25 years or older (odds ratio [OR], 0.87; 95% confidence interval [CI], 0.79 to 0.96) (Table 3). All other associations from the bivariate analysis remained in the adjusted analysis.

**TABLE 3.** Service Delivery and Clinical Care Characteristics of Clients Receiving Any Postabortion Contraception^a^ at the Time of Abortion Care in 10 Countries, Bivariate and Adjusted Analysis, 2011–2015

	Accepted Postabortion Contraception (n=709,192) No. (%)	Did Not Accept Postabortion Contraception (n=212,726) No. (%)	Bivariate Analysis (n=921,918)		Adjusted Analysis^b^ (n=816,880)	
			OR	95% CI	AOR	95% CI
Facility sector						
Private	187,124 (26)	45,831 (22)	Reference		Reference	
Public	522,068 (74)	166,895 (78)	0.77***	(0.76, 0.78)	0.88	(0.71, 1.10)
Ipas-trained provider						
Yes	449,492 (63)	102,044 (48)	1.88***	(1.86, 1.90)	1.37***	(1.20, 1.57)
No	259,700 (37)	110,682 (52)	Reference		Reference	
Client age						
≤19	48,725 (7)	19,540 (9)	0.71***	(0.70, 0.72)	0.87**	(0.79, 0.96)
20–24	215,964 (30)	66,731 (31)	0.92***	(0.91, 0.93)	0.96	(0.92, 1.01)
≥25	444,503 (63)	126,455 (59)	Reference		Reference	
Gestational age, weeks^c^						
≤12	663,391 (96)	178,937 (91)	Reference		Reference	
≥13	25,777 (4)	17,894 (9)	0.39***	(0.38, 0.40)	0.73***	(0.65, 0.81)
Missing	20,024	15,895				
Type of abortion^c^						
Induced abortion	438,742 (65)	79,965 (41)	Reference		Reference	
PAC	238,504 (35)	113,977 (59)	0.38***	(0.38, 0.39)	0.55***	(0.45, 0.67)
Other/missing	31,946	18,784				
Abortion method^c^						
MVA/EVA	514,163 (75)	150,299 (72)	Reference		Reference	
Medical abortion	151,801 (22)	36,366 (17)	1.22***	(1.20, 1.24)	1.20*	(1.01, 1.43)
D&C/D&E/other	20,645 (3)	22,459 (11)	0.27***	(0.26, 0.27)	0.34***	(0.27, 0.42)
Other/missing	22,583	3,602				

Abbreviations: AOR, adjusted odds ratio; CI, confidence interval; D&C, dilation and curettage; D&E, dilation and evacuation; EVA, electric vacuum aspiration; MVA, manual vacuum aspiration; OR, odds ratio; PAC, postabortion care.

* *P*<.05; ** *P*<.01; *** *P*<.001.

a Sterilization excluded.

b Adjusted odds ratios control for country variation and cluster on facility (using non-missing data only).

c Other/missing data were not included in the calculation of percentages.

Women under age 20 were less likely to choose a method than women 25 years or older.

#### Factors Associated With Choice of a LARC

In the adjusted model, acceptance of a LARC method was more likely if a client received care in a public facility, was 25 years of age or older, sought an induced abortion (compared with those who sought PAC services), or had a vacuum aspiration abortion (MVA or EVA) compared with medical abortion (Table 4). Adolescents and young women were significantly less likely to select a LARC method than women 25 years or older (age ≤19 years: OR, 0.59; 95% CI, 0.52 to 0.67; age 20–24 years: OR, 0.68; 95% CI, 0.63 to 0.73).

**TABLE 4.** Service Delivery and Clinical Care Characteristics of Clients Receiving a LARC at the Time of Abortion Care in 10 Countries, Bivariate and Adjusted Analysis, 2011–2015

	Accepted LARC (n=113,694) No. (%)	Did Not Accept LARC (n=595,498) No. (%)	Bivariate Analysis (n=709,192)		Adjusted Analysis^a^ (n=638,666)	
			OR	95% CI	AOR	95% CI
Facility sector						
Private	36,785 (32)	150,339 (25)	Reference		Reference	
Public	76,909 (68)	445,159 (75)	0.71***	(0.70, 0.72)	1.42**	(1.16, 1.74)
Ipas-trained provider						
Yes	72,856 (64)	376,636 (63)	1.04***	(1.02, 1.05)	0.97	(0.85, 1.11)
No	40,838 (36)	218,862 (37)	Reference		Reference	
Client age, years						
≤19	6,621 (6)	42,104 (7)	0.74***	(0.72, 0.76)	0.59***	(0.52, 0.67)
20–24	29,279 (26)	186,685 (31)	0.74***	(0.73, 0.75)	0.68***	(0.63, 0.73)
≥25	77,794 (68)	366,709 (62)	Reference		Reference	
Gestational age, weeks^b^						
≤12	107,155 (96)	556,236 (96)	Reference		Reference	
≥13	3,899 (4)	21,878 (4)	0.93***	(0.89, 0.96)	1.04	(0.93, 1.27)
Missing	2,640	17,384				
Type of abortion^b^						
Induced abortion	78,926 (72)	359,816 (63)	Reference		Reference	
PAC	30,918 (28)	207,586 (37)	0.68***	(0.67, 0.69)	0.44***	(0.38, 0.50)
Other/missing	3,850	28,096				
Abortion method^b^						
MVA/EVA	83,998 (80)	430,165 (74)	Reference		Reference	
Medical abortion	19,382 (18)	132,419 (23)	0.75***	(0.74, 0.76)	0.52***	(0.44, 0.63)
D&C/D&E/other	2,204 (2)	18,441 (3)	0.61***	(0.59, 0.64)	0.86	(0.68, 1.08)
Missing	8,110	14,473				

Abbreviations: AOR, adjusted odds ratio; CI, confidence interval; D&C, dilation and curettage; D&E, dilation and evacuation; EVA, electric vacuum aspiration; LARC, long-acting reversible contraceptive; MVA, manual vacuum aspiration; OR, odds ratio; PAC, postabortion care.

* *P*<.05; ** *P*<.01; *** *P*<.001.

a Adjusted odds ratios control for country variation and cluster on facility (using non-missing data only).

b Other/missing data were not included in the calculation of percentages.

Adolescents and young women were significantly less likely to select a LARC than women 25 years or older.

## DISCUSSION

### Key Results

Overall, in the nearly 1,000,000 abortion cases treated in 10 countries of sub-Saharan Africa and Asia, we found high rates of postabortion contraception acceptance among the clients, but adolescents were significantly less likely than women 20 years of age or older to choose to use contraception. Women 15–19 years old and those 20–24 years old had similar uptake of LARC methods, and women 25 or older had significantly higher odds of receiving a LARC method than these younger women. Women cared for by a provider who participated in an Ipas-supported training were significantly more likely to choose a postabortion contraceptive method, but differences by age remained. While contraceptive uptake was high in our analysis (77% of women left the health facility with a contraceptive method), our findings underscore that young women, especially adolescents, may not have access to the full range of contraceptive methods for which they are medically eligible,^24^ and additional attention to this group is needed to enable them to prevent unintended pregnancy.

Young women, especially adolescents, may not have access to the full range of contraceptive methods for which they are medically eligible.

### Trained Providers and Clients' Contraceptive Uptake

Abortion care clients of providers who participated in an Ipas-supported training were significantly more likely to select a postabortion contraceptive method, but no differences in LARC acceptance by clients of Ipas-trained versus non-Ipas-trained providers were observed. While we do not have data on providers who did not directly participate in the trainings or who did not receive post-training follow-up, non-Ipas-trained providers may not have prioritized postabortion contraception, or they may have perceived their counseling or clinical skills as insufficient. Another possibility is that they may have held misconceptions about contraceptive methods during the postabortion period. For example, non-Ipas-trained providers may have been unaware that women who select medical abortion and also wish to use a hormonal method of contraception (i.e., pills, injectables, or implants) may start the method on day 1 of the abortion medication regimen, per WHO guidelines.^22^^,^^24^ The findings suggest a need for further reinforcement of these guidelines, as well as a need to emphasize counseling and LARC method provision as part of training and follow-up and to implement approaches for updating providers who do not attend trainings.

### Pregnancy Gestation and Clients' Contraceptive Uptake

Women with gestations of 13 weeks or more were significantly less likely to choose a contraceptive method than women presenting at 12 weeks or less, a troubling finding since adolescents and young women were disproportionately represented among clients seeking care in later stages of pregnancy.

Women with gestations of 13 weeks or more were significantly less likely to choose a contraceptive method than women presenting at 12 weeks or less.

Abortion care clients in later pregnancy gestations are more likely to obtain services in hospital settings than primary-level sites. Hospital environments present special challenges for postabortion contraception, with several studies reporting that women obtaining abortion care in hospitals are less likely to receive any contraception than those in primary health centers or clinics.^18^^,^^25^ A variety of factors likely contribute to this finding, including the multiple health care workers attending a single client in a hospital, logistical difficulties in ensuring that contraceptive commodities are routinely available in the abortion treatment area, the perception that contraceptive provision is not a physician's task, provider workloads that prioritize more acute care, and administrative barriers to linking 2 different services. With their lower uptake of contraception, clients seeking abortion at 13 weeks or greater gestation therefore represent a group at risk of subsequent unintended pregnancy.

However, in our study, clients who received contraception were more likely to obtain a LARC method in public facilities than private sites. Larger, public hospitals may be more likely to have had trained and experienced providers with access to LARC supplies and commodities than smaller, private facilities; many primary-level providers who participated in training were new to abortion care and may not have had experience in LARC provision. These findings underscore the importance of access to an array of contraceptive methods and a trained provider across facility sectors and levels for all women seeking abortion care.

### Type of Abortion Care and Clients' Contraceptive Uptake

PAC clients include women who require treatment of abortion complications from induced abortions obtained elsewhere; women who have used medical abortion outside of a health facility and present with bleeding; those presenting for miscarriage management of wanted pregnancies and who wish to have another pregnancy soon; and other miscarriage patients who want to delay a subsequent pregnancy. In the adjusted model, women seeking induced abortion were much more likely to choose a contraceptive method than those obtaining PAC services. This finding could partly be due to provider bias if they assumed that most PAC clients had had a miscarriage and wanted to become pregnant again soon. In addition, providers treating patients with severe abortion complications such as septic abortion and shock often prioritize immediate medical needs. While a significant amount of research has been conducted on the uptake of postabortion contraception by PAC clients, a better understanding of women's needs and preferences at the time of care and competing provider constraints could contribute to improved quality.^14^^,^^26^^,^^27^ In addition, while most induced abortion clients experience unintended pregnancies and want to prevent a subsequent one, some may want to become pregnant again.

These diverse circumstances underscore the importance of counseling that incorporates clients' fertility preferences and offers adolescents and women their choice of contraceptive methods. Induced abortion clients who accepted contraception were highly significantly more likely to choose a LARC method than PAC clients accepting contraception. Our data do not allow us to determine precise reasons for this finding, but they suggest that PAC clients who want to use contraception may prefer short-acting methods until they recover from the incomplete abortion and are ready to make decisions about pregnancy. Furthermore, PAC clients with septic abortion are not medically eligible for IUD insertion at the time of the abortion, which may also contribute to the finding.

### Method of Abortion and Clients' Contraceptive Uptake

Differences in contraceptive uptake by abortion technology have also been found in other studies using monitoring data from Ipas-supported facilities, with similar or higher acceptance of any contraceptive method with medical abortion compared with MVA/EVA but lower LARC uptake.^18^^,^^25^ Women receiving induced abortion and contraception in Indian NGO clinics found that acceptance of any method was similar for MVA and medical abortion clients within 2 months following services, although method mix varied. Women who had an MVA were more likely to have had an IUD or sterilization, while medical abortion clients mainly chose IUDs and condoms. This likely reflects clinical protocols for MVA clients that permit sterilization or IUD insertion at the time of the procedure.^28^ Health facilities should stock contraceptive implant commodities and abortion providers should be trained in their insertion on the first day of a medical abortion if the adolescent or woman desires this method.

### Uptake of LARC Methods

Provision of LARC methods to adolescents has received increasing attention in recent years. In the United States, both the American Academy of Pediatrics and the American College of Obstetricians and Gynecologists have issued statements on the safety and appropriateness of IUDs and implants for adolescents who desire these methods.^29^^,^^30^ WHO's medical eligibility criteria indicate that age is not a contraindication to LARC provision.^31^ Research on contraceptive services that incorporated information on method types and their risks and benefits, especially for LARCs, and provided methods at no cost to the client demonstrated high uptake of LARCs among adolescents. In a follow-up period that lasted 2 to 3 years, adolescents in the study had lower rates of pregnancy, births, and abortion compared with all U.S. adolescents.^32^ A systematic review of research on IUDs in the United States found that the method is safe and effective for young women and adolescents.^33^

The Ipas training curriculum, clinical guidelines, and post-training technical support to providers and facilities are consistent with WHO's guidance on abortion care and postabortion contraception and more recent published evidence. These state that all contraceptive methods may be provided to women at the time of an uncomplicated first- or second-trimester abortion procedure performed with vacuum aspiration or medical abortion, according to their medical eligibility.^22^^,^^24^ This recommendation includes LARC methods consisting of IUDs and implants. For women treated for septic abortion who select an IUD, insertion should be delayed until the infection is resolved. As previously noted, hormonal methods can be started on day 1 of administration of a medical abortion regimen. For medical abortion clients who wish to have an IUD, providers should first be certain that the woman is no longer pregnant and that the abortion was successful.^24^ This usually precludes IUD insertion at the time of a medical abortion unless the woman completes the abortion at the facility; women selecting medical abortion and desiring an IUD should be offered a short-acting method to prevent pregnancy until they are able to return to the facility for the insertion. This guidance should be a part of routine training and supervision of abortion providers so that they apply the latest clinical evidence in their practices, especially for young clients.

The provision of LARCs to adolescents, especially those who have not given birth, remains a relatively new concept in many countries in Africa, Asia, and Latin America.^34^^,^^35^ The lack of availability of certain methods also limits young women's choices. One important example is India, where implants are unavailable and injectables have only recently been approved by the national Ministry of Health. India represented a large proportion of cases in our analysis, and women and adolescents there had access to only 1 LARC method, IUDs, following their abortion.

### Strengths and Limitations

Our analysis had the advantages of including a large number of abortion care cases in a variety of service delivery environments in 10 countries. Recordkeeping on client information was of good quality with generally low rates of missing data, and data were available on individual clients. Literature focused specifically on increasing use of LARCs among adolescents in developing countries is quite limited.^34^ Published research on interventions to provide LARCs to adolescents following abortion care in these countries is virtually unavailable. Our analysis provides new evidence on an intervention that included provision of LARC methods to adolescent abortion clients.

Limitations of our analysis and findings include the fact that data were not available before Ipas began programmatic support due to the initial poor quality of abortion care log books in most settings. Resource limitations did not permit data collection in comparison facilities that had not received specialized abortion training for providers nor site upgrades. Furthermore, log book data were recorded by providers themselves and therefore had the potential for error or underreporting, especially given the stigma associated with abortion care.

Abortion care log book entries rarely include women's fertility desires, and even if noted, the data are unreliable. Women's preferences about if and when they wish to become pregnant again can be collected through other methodologies such as client exit interviews or observation of provider-client interactions during service delivery. Ideally, log book data on contraceptive uptake would be paired with clients' responses on the quality of services, contraceptive method preferences, appropriateness of information offered, and their fertility plans.

Our data did not include an important group, women who use medical abortion on their own or with the help of individuals outside a clinical setting. Very little is known of their postabortion contraceptive patterns; the methodological challenges of reaching these women are daunting.^36^ With the increasing availability of mifepristone and misoprostol in pharmacies and medicine shops and through the Internet and other outlets, researchers will need to develop approaches to learn if and how women, especially adolescents, access contraception following medical abortion obtained outside a health facility setting.

## PROGRAMMATIC RECOMMENDATIONS

Although this study found relatively high rates of postabortion contraceptive acceptance, gaps remain, especially for adolescents. Any programmatic recommendations to improve postabortion contraception as part of facility-based abortion care for adolescents and young women must maximize the available evidence from other sexual and reproductive health interventions. “Youth-friendly” sexual and reproductive health services are common but often suffer from fragmentation and spotty implementation.^37^ The evidence points to the value of youth-friendly services that include health worker training and creation of welcoming environments for young people, integrated with community outreach and demand creation efforts to young people and community members.^37^^–^^39^ Adolescents are not a homogenous group, and comprehensive abortion care services that seek to improve postabortion contraceptive provision, should be assessed to determine if they meet the needs of subgroups of adolescent clients (e.g., married/unmarried, in/out-of-school, those in humanitarian settings).

Although postabortion contraceptive uptake was relatively high among the population studied, gaps remain, especially for adolescents.

Our recommendations to improve postabortion contraception for adolescents and young people receiving health facility care include the following:

- Update policies and protocols to eliminate barriers such as requirements that women and adolescents have to be married or have parental or spousal consent for contraceptive services, and ensure that hormonal methods are indicated as appropriate on the same day of medical abortion
- Implement provision of a wide choice of contraceptive methods for adolescents and young women to select from, including LARC methods
- Ensure that contraceptive methods are available in all locations within a facility where induced abortion services and PAC are offered
- Train providers, clinic managers, and facility staff to improve counseling and client interactions, upgrade clinical skills on postabortion contraceptive methods, and implement efforts to reduce stigma
- Consider post-training follow-up support of providers and facilities to reinforce new skills and resolve obstacles to care
- Upgrade facilities to ensure privacy, especially for adolescents, and ensure confidentiality of client interactions and records
- Reduce or eliminate out-of-pocket costs of all contraceptive methods, especially for adolescent clients
- Include frequent client and community interviews to determine the preferences of young women and adolescents, and assess the quality of postabortion contraceptive care they receive

While our study finds that important progress has been achieved in improving postabortion contraceptive care in Ipas-supported facilities, many health facilities in developing countries still do not offer comprehensive services. This gap is especially tragic for young women and adolescents who are most vulnerable to unintended pregnancy and unsafe abortion. Increased contraceptive use, including after abortion, is critical to their improved sexual and reproductive health and well-being.

## Notes

**Peer Reviewed**

**Funding:** Ipas and Ipas Development Foundation.
